# Using Transcranial Magnetic Stimulation to Test a Network Model of Perceptual Decision Making in the Human Brain

**DOI:** 10.3389/fnhum.2020.00004

**Published:** 2020-01-24

**Authors:** Bruce Luber, David C. Jangraw, Greg Appelbaum, Austin Harrison, Susan Hilbig, Lysianne Beynel, Tristan Jones, Paul Sajda, Sarah H. Lisanby

**Affiliations:** ^1^Department of Psychiatry and Behavioral Science, Duke University School of Medicine, Durham, NC, United States; ^2^Department of Biomedical Engineering, Columbia University, New York, NY, United States

**Keywords:** transcranial magnetic stimulation, perceptual decision making, lateral occipital complex, object discrimination, chronometry

## Abstract

Previous research has suggested that the lateral occipital cortex (LOC) is involved with visual decision making, and specifically with the accumulation of information leading to a decision. In humans, this research has been primarily based on imaging and electroencephalography (EEG), and as such only correlational. One line of such research has led to a model of three spatially distributed brain networks that activate in temporal sequence to enable visual decision-making. The model predicted that disturbing neural processing in the LOC at a specific latency would slow object decision-making, increasing reaction time (RT) in a difficult discrimination task. We utilized transcranial magnetic stimulation (TMS) to test this prediction, perturbing LOC beginning at 400 ms post-stimulus onset, a time in the model corresponding to LOC activation at a particular difficulty level, with the expectation of increased RT. Thirteen healthy adults participated in two TMS sessions in which left and right LOC were stimulated separately utilizing neuronavigation and robotic coil guidance. Participants performed a two-alternative forced-choice task selecting whether a car or face was present on each trial amidst visual noise pre-tested to approximate a 75% accuracy level. In an effort to disrupt processing, pairs of TMS pulses separated by 50 ms were presented at one of five stimulus onset asynchronies (SOAs): −200, 200, 400, 450, or 500 ms. Behavioral performance differed systematically across SOAs for RT and accuracy measures. As predicted, TMS at 400 ms resulted in a significant slowing of RT. TMS delivered at −200 ms resulted in faster RT, indicating early stimulation may result in priming and performance enhancement. Use of TMS thus causally demonstrated the involvement of LOC in this task, and more broadly with perceptual decision-making; additionally, it demonstrated the role of TMS in testing well-developed neural models of perceptual processing.

## Introduction

The human brain is adept at interpreting visual input with a remarkable ability to process features, objects, and scenes, rapidly performing complex categorizations. These abilities are at the core of human visual cognition, and there has been a concerted effort from cognitive neuroscientists to elucidate the underlying neural mechanisms that enable perceptual decision making (PDM; for reviews, see Kelly and O’Connell, 2015; Gold and Stocker, 2017).

Previous research addressing perceptual decision-making processes has frequently focused upon instances when discrimination of visual objects is difficult. For example, studies by Heekeren et al. (2004) presented images of faces and houses masked by varying levels of visual noise to investigate the cortical mechanisms underlying PDM with functional magnetic resonance imaging (fMRI). Their results demonstrated that portions of the dorsal lateral prefrontal cortex activate more in response to easy-than-difficult decisions, and covary with the difference in responses from the face- and house-selective regions of the ventral temporal cortex, while also predicting behavioral performance in the categorization task. These and similar findings (Shadlen and Newsome, 2001; Paulus et al., 2002; Grinband et al., 2006; Kahnt et al., 2011) support the notion that spatially-distributed neural networks compare information collected from low-level sensory areas to perform complex PDM.

The effort to elucidate neural mechanisms of PDM has been supported by the powerful combination of electrophysiological and hemodynamic measures of brain function with complementary spatial and temporal sensitivity (e.g., Ales et al., 2013; Di Russo and Pitzalis, 2014). In one particularly fruitful line of research, Philiastides et al. (2006) incorporated electroencephalography (EEG) and fMRI collected during variants of a face/car discrimination task to characterize distributed networks that activate in sequence during PDM. Through a series of three studies (Philiastides and Sajda, 2006, 2007; Philiastides et al., 2006), these authors utilized single-trial logistic regression on EEG, drift diffusion modeling of behavioral data, and EEG-informed fMRI analysis to ascertain the cortical origins of three temporally specific neural networks sensitive to different elements of the task parameterization.

In their visual task, participants discriminated face from car images that were degraded in perceptual clarity through scrambling of spatial phase. In the first two studies, 60-channel EEG recorded during performance of the task was analyzed on a single trial basis using logistic regression to maximally distinguish face and car trials (Philiastides and Sajda, 2006; Philiastides et al., 2006). Psychometric functions relating to performance accuracy and coherence level were statistically indistinguishable from neurometric functions relating the strength of classification to coherence levels, suggesting the EEG reflected the workings of the neural substrate of the categorization. The best matches of these functions occurred in an early latency window, centered on 170 ms from stimulus onset, which corresponds to the N170 ERP component well- known to be involved with stimulus categorization, and a later window, beginning after 300 ms latency, which formed even better matches with the performance data. In fact, both the onset latency and the duration of the later EEG component increased with discrimination difficulty (with increasing difficulty pushing its onset past 400 ms), relationships not found in the early component. Further, the early component was just as active when evoked during a simple red/green discrimination, while the late component was only evoked when the more difficult degraded face/car categorization was made. Implementing a drift-diffusion model, using behavioral performance to link the accumulation of information over time to decision choices, it was found that the estimated drift rate in the model was strongly correlated with the strength of discrimination estimated from the EEG data of the late (but not the early) component. Furthermore, a third component, peaking around 220 ms, was also identified, whose activity was found to be closely bound to the activity of the late component and to stimulus difficulty: its amplitude was inversely proportional both to the stimulus evidence in the model and to the onset of the late component. Overall, the evidence of these studies indicated three components of neural activity: an early one involved in initial perceptual processing, and two later ones closely linked to PDM.

**Figure 1:**
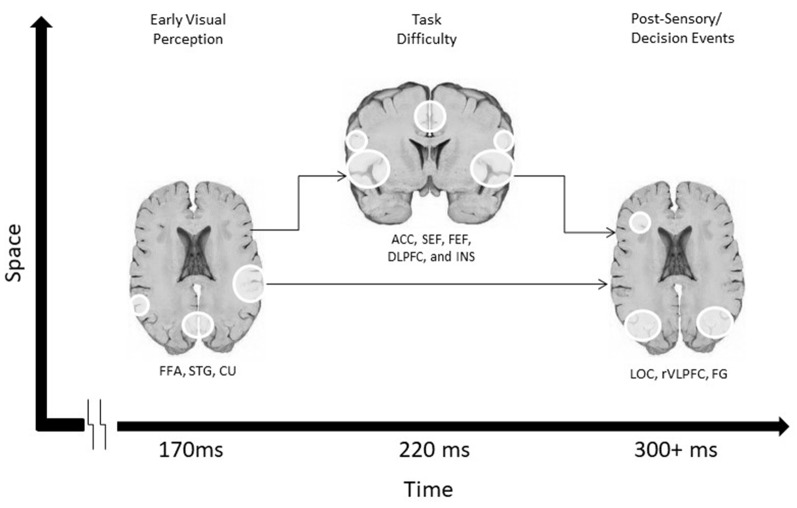
A schematic Spatio-temporal diagram of the three networks involved in visual discrimination in Philiastides and Sajda (2007).

A third study from this group utilized fMRI to ascertain the cortical origins of each of the three temporally-specific EEG components (Philiastides and Sajda, 2007). Using the previous EEG results as fMRI regressors, they identified a Spatio-temporal cascade of activity in three spatially-distributed networks, with contributions from the fusiform face area and superior temporal sulcus associated with the early component, a network of mainly frontal attention- related areas mediating the difficulty-dependent second component, and the involvement of the lateral occipital cortex (LOC) with the later component. This fMRI study, therefore, tied together findings from the other two studies to link the spatial and temporal patterns of activity in networks underlying decision-making in uncertain conditions by correlating behavioral performance with network activity (Figure 1).

While these studies provide strong evidence towards the involvement of discrete brain networks in different stages of PDM, their findings are correlational and do not provide definitive evidence of causal brain-behavior relationships. In contrast to EEG and fMRI, transcranial magnetic stimulation (TMS) can be used to establish such causal links, given its ability to selectively perturb neural information processing and measure the effects on behavior. In particular, the exacting psychophysical, electrophysiological and imaging work of Philiastides, Sajda and colleagues lends itself to a very specific test of their dynamic neurophysiological model. Namely, that a pair of TMS pulses, applied during a time window beginning at 400 ms after the stimulus onset, would inject neural noise during the sensitive period related to the difficult face/car PDM, thus slowing down discrimination processing, resulting in a longer reaction time (RT). The timing parameters used in this prediction were carefully based on the findings of Philiastides et al. (2006) in three ways. First, we took advantage of the relationship between discrimination difficulty and onset latency of the late component found by Philiastides et al. (2006) in which latency increases with difficulty. We did this by titrating phase coherence using an adaptive staircase in each subject to a specific accuracy level of 79% for both face and car stimuli, a phase coherence level could be applied that would be expected to push the onset the latency of the late component to an approximate time of 400 ms, based on the temporal relationship of phase coherence with late component onset latency found previously (Philiastides and Sajda, 2006; Philiastides et al., 2006). Second, the timing of the pair of TMS pulses, separated by 50 ms, was chosen to fit the window duration that best discriminated the late component in the single-trial EEG analyses (Philiastides et al., 2006). The timing of our paired pulses are based in part on the previous literature using paired-pulse TMS (ppTMS) on LOC during visual tasks, in which TMS performance effects could be found and discriminated from surrounding time windows with paired pulses separated by 100 ms (Mullin and Steeves, 2011), 50 ms (Ellison and Cowey, 2007), 40 ms (Pitcher et al., 2007), down to 10 ms (Pitcher et al., 2012). We were trying to affect a visual discrimination process that is extended over time, based on the EEG work possibly for 50–100 ms, so we chose pulses that would occur around the onset of the process and 50 ms into it, with the 50 ms time chosen based on the duration of the EEG analysis window that best discriminated the process. Third, based on the relationship of EEG activation and drift rate in the diffusion model of behavioral data during the late component (Philiastides et al., 2006), we expected the random neural excitement added by TMS beginning at 400 ms would approximate the addition of noise to the diffusion model, resulting in slower processing and thus a lengthening of RTs.

In accordance with this prediction, participants in the present study performed a speeded version of the face/car task with ppTMS applied with a 50 ms interstimulus interval introduced at a number of latencies spanning from 200 ms before stimulus presentation, to 500 ms post-stimulus. ppTMS was applied to the LOC, the major source of the late component activity in the Philiastides and Sajda MRI study (Philiastides and Sajda, 2007). It was hypothesized that this stimulation during the active phase of PDM would result in impaired performance relative to stimulation at 500 ms, a latency the previous work indicated was past the completion of the late component. As such, the current design utilized a chronometric approach to perturb neural function across the range of times specifically identified as important for PDM, providing a causal test of these correlational relationships and the Philiastides et al. (2006) model, as well as providing causal evidence for the involvement of LOC in post-sensory decision making more generally.

## Materials and Methods

### Participants

Fifteen healthy volunteers were recruited and provided written informed consent for the study, approved by the Institutional Review Board of the Duke University Medical Center. Two dropped out for scheduling reasons, leaving 13 completing the full study. These 13 individuals (five females) had a mean age of 24.6 ± 2.8 years. Participants had normal, or corrected-to-normal, vision. Participants were excluded if they had a current or past Axis I psychiatric disorder (MINI International Neuropsychiatric Interview, 5.0.0 DSM-IV (Sheehan et al., 2006), or a history of neurological disease (TMS Adult Safety Screen: Keel et al., 2001). All participants were screened for substance abuse with urine drug screens and women of childbearing capacity were screened with urine pregnancy tests. Following the screening, subjects were introduced to the visual discrimination task, returning for an MRI session and two TMS sessions within the next 2 weeks.

### Stimuli

**Figure 2:**
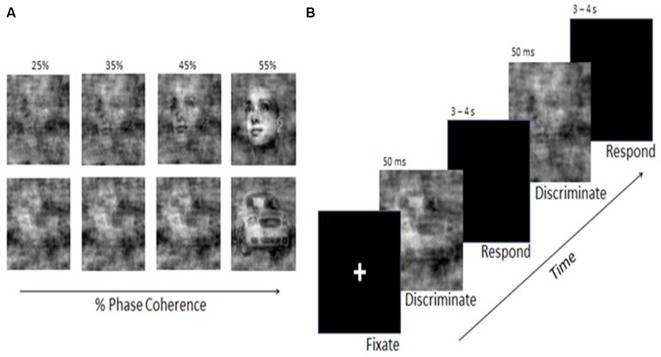
**(A)** Examples of face and car stimuli with various levels of noise added by manipulation of the phase coherence in the image. **(B)** Schematic illustration of the Visual Discrimination Task. Face or car images appeared for 50 ms, followed by a response interval of 3–4 s. Phase coherence varied across trials according to a staircase schedule and determined by each individual’s performance.

A set of 12 faces (chosen from the Max Planck Institute face database^1^) and 12 car images were used. All images were rendered in grayscale with 8 bits/pixel, were 512 × 512 pixels in size, and were equated for spatial frequency, luminance, and contrast (for a more complete description, see Philiastides et al., 2006). Stimuli were presented on a Dell UltraSharp 20.1″ monitor at a distance of 100 cm, using E-Prime software (Psychological Software Tools, Pittsburgh, PA) to control the stimulus display. Visual stimuli were presented in the central visual field, with each image subtending 33 × 22° of visual angle. The phase spectra of the face and car images were manipulated to generate sets of stimuli with varying degrees of phase coherence (Figure 2A).

### Visual Discrimination Task

Participants were seated facing the monitor. Using a Cedrus RB-830 Response Pad (San Pedro, CA, USA), they were instructed to respond as quickly and as accurately as possible by pressing one of two buttons to indicate whether the image was a face or a car. The order of presentation for the car and face stimuli was randomized across trials. Stimuli were presented for 50 ms, followed by a blank screen over an inter-trial interval that was randomly selected with a uniform distribution between 3,000 and 4,000 ms (Figure 2B).

### Psychometric Staircase

At the beginning of each session, participants performed the task until their performance accuracy became stable at 79% for both stimulus types. This served two purposes: to reduce performance variability between individuals, and, more importantly, to push subjects to perform in the more difficult range of coherence levels that were previously shown to correspond to the onset of the late component at approximately 400 ms (Philiastides and Sajda, 2006; Philiastides et al., 2006).

This was achieved using a staircasing algorithm (Levitt, 1970), which dynamically changed the image coherence in successive stimuli, independently for faces and cars. To that end, two interleaved staircase functions were run across a block of trials: one containing only faces and the other containing only cars, with trials from the two staircases interspersed randomly. Both staircases were begun at the easiest (i.e., most discriminable) level used: 50% phase coherence. If the participant answered three consecutive trials from one staircase correctly, the difficulty of that staircase increased (i.e., decreased coherence in the next stimulus), while a single incorrect response decreased the difficulty level one step. This “three-up, one-down” procedure theoretically converges at a participant performance level of 79.4% correct. Each time the change in difficulty changed sign it was considered a “reversal.” Before the 4th reversal, the difficulty changed in increments of 10% phase coherence; between the 4th and 10th reversals, in increments of 3%; after the 10th reversal, in increments of 1%. The paradigm ended after both staircases had completed 25 reversals, allowing subjects to reach constant performance. For each staircase, the coherence levels over the last 10 reversals were averaged to estimate threshold performance.

### MRI Acquisition and Analysis

MRIs were obtained for use in TMS targeting. MRI scanning was conducted on a 3T General Electric scanner using an 8 hBrain coil configuration, and whole-brain anatomical scans were acquired using a 3D T1-weighted echo-planar sequence (TR = 8.208 ms, TE = 3.22 ms, FOV = 240*240, slice thickness = 1.6 mm). During this scan, the participants viewed a blank screen. Anatomical images were skull-stripped using FMRIB Software Library (FSL v.5.0). The left and right LOC coordinates found in previous group fMRI analyses using the face/car discrimination task (Philiastides and Sajda, 2007; left LOC: −42, −88, −10, right LOC: 46, −86, −8; Montreal Neurological Institute Template) were registered on each individual brain using FSL affine linear regression tool to be used as TMS targets.

### TMS Sessions

Two TMS sessions were run on separate days, each lasting approximately 2 h. Each session consisted of the staircasing procedure to obtain the coherence levels for each image type. This was followed by 6 blocks of visual discrimination trials, with TMS targeted to either left or right LOC. The other LOC site was targeted in the second session, with site order counterbalanced across participants. While previous studies have found both left and right LOC to be active during visual discrimination tasks (Philiastides and Sajda, 2007; Ales et al., 2013), left and right sites were stimulated separately here in order to evaluate laterality effects.

TMS was delivered using a Cool-B65 Butterfly figure-8 coil powered by a MagProX100 the stimulator (MagVenture, Farum, Denmark). The coil was positioned using SmartMove, a robotized TMS coil positioning system (ANT, Enschede, Netherlands) allowing 300 ms recovery and 1–3 mm precision. Stimulus intensity was set at 100% of the participant’s resting motor threshold, collected at the beginning of the first TMS session and defined as the minimum intensity needed to evoke motor potentials of at least 50 μV recorded *via* EMG from the first dorsal interosseus muscle of the right hand in at least 5 out of 10 stimulations (Rossi et al., 2009).

One-sixth of the task trials in a block of trials were no-TMS trials in which no TMS was delivered, and the rest were TMS trials. In each TMS trial, paired TMS pulses separated by 50 ms were delivered. Past research has indicated ppTMS can be used to disrupt visual processing in LOC (Ellison and Cowey, 2007; Pitcher et al., 2008; Mullin and Steeves, 2011). The 50 ms timing of the pulses was chosen to disrupt processing over a similar time range as the best window duration to capture the activation and psychophysiological relationships of the late component in single-trial EEG analyses (Philiastides et al., 2006). The first of the two pulses was time-locked to the onset of the visual stimulus at one of five stimulus onset asynchronies (SOAs): −200, 200, 400, 450 or 500 ms. The choice of SOA (or no-TMS) in a given trial was made pseudo-randomly, and there were 162 trials for each of the six conditions across the session.

ppTMS applied at 500 ms SOA was defined as the control condition, given that the previous research indicated that the processing associated with the late component at LOC was expected to be completed at this time (Philiastides and Sajda, 2006; Philiastides et al., 2006). Such a temporal control condition has been used in the field since its inception (e.g., Amassian et al., 1989), and by our own group in previous visual studies (Matthews et al., 2001; Luber et al., 2007). This control condition presents several advantages compared to using an active control site or a passive sham condition: (1) the different SOAs can be randomly interspersed within the same block of trials, with the subject having little awareness of their difference and no ability to predict them; (2) these conditions feel the same to the participant; and (3) the different conditions stimulate the same nervous tissue. Moreover, a second control site is unnecessary in the present case, as we are specifically testing the prediction that TMS to LOC at a specific time (relative to another) will slow RT.

### Analysis

Median RTs (in correct trials) and accuracy were calculated for each stimulus type at each SOA. A 2 × 2 × 6 repeated-measures ANOVA was performed for each measure, with factors of Site (left, right), Stimulus type (face, car), and SOA (−200, 200, 400, 450, 500, no-TMS). However, MANOVAs were substituted because tests for sphericity produced Greenhouse-Geisser epsilon <0.70 in both cases. Planned analyses were performed for RT and accuracy measures to test the prediction that TMS at the 400 and 450 ms SOAs (Bonferroni-corrected) had a deleterious effect on performance relative to 500 ms. Exploratory *post hoc* tests were done between the 500 ms condition and: (1) the no-TMS condition, to provide evidence that TMS at 500 ms had no effect on performance; (2) the 200 ms condition, to observe any performance effects at the latency of the second component, which was related to difficulty and expected to be active at the stimulus coherence levels employed (Philiastides and Sajda, 2006); and (3) the −200 ms condition, to observe whether a pulse prior to the onset of the trial affected performance, as has occurred in other TMS studies (e.g., Grosbras and Paus, 2003). Effect sizes were estimated using Cohen’s *d*, calculated in a repeated-measures situation as the *t*-value obtained, divided by the square root of the degrees of freedom (Rosenthal and Rosnow, 1991).

## Results

### Coherence Thresholds

The titrated coherence thresholds for face and car stimuli were stable across the two sessions and were similar for both types of stimuli. The group mean coherence for cars (34.0% ± 6.3) was higher than for faces (29.5% ± 5.6), but not significantly so. A 2 × 2 repeated-measures ANOVA on the threshold estimates, looking across the two sessions and the two stimulus types (face, car) showed no significant main effect for either factor. There was a significant Session by Stimulus-Type interaction (*F*_(1,12)_ = 7.0, *p* < 0.02), due to a decrease in average coherence needed for cars between the first and second sessions, although a *post hoc* paired *t*-test was not significant for this difference. The titration procedure proved successful, in that the coherence levels used for each participant produced accuracy levels close to the expected staircase accuracy during the experimental sessions for both face (group mean and SD in the non-TMS condition: 75.6% ± 14.6) and car stimuli (75.6% ± 12.2).

### No-TMS vs. 500 ms SOA Conditions

To test the reliability of 500 ms SOA as a control condition, we compared performance obtained in this condition to no-TMS condition performance. There were no differences between these two conditions in either accuracy (*t*_(12)_ = 1.9, *p* = 0.29) or RT (*t*_(12)_ = 1.2, *p* = 0.26). This lack of difference provides validation for the use of 500 ms SOA as a control condition, as expected from the EEG data of Philiastides and Sajda (2006) and Philiastides et al. (2006), in which activation related to the late component was complete by 500 ms.

### Reaction Time

**Figure 3:**
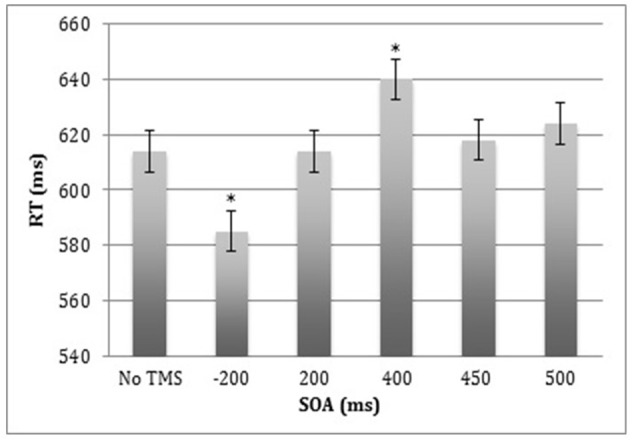
Median reaction time (RT) for correct trials averaged across the left and right lateral occipital cortex (LOC) stimulation sites and the face and car stimulus types. Error bars represent standard errors.

Behavioral analyses revealed a significant effect of SOA (*F*_(5,8)_ = 16.5, *p* < 0.0005), but no main effects of Site or Stimulus Type, and no significant interactions (Figure 3). Bonferroni-corrected analyses showed that when ppTMS was applied at 400 ms SOA, RT was slower relative to ppTMS applied at 500 ms SOA (*t*_(12)_ = 2.9, *p* < 0.015; Cohen’s *d* = 0.84). However, when TMS was applied at −200 ms SOA, RT was significantly faster compared to the 500 ms SOA (*t*_(12)_ = 4.6, *p* < 0.0005; Cohen’s *d* = 1.33).

### Accuracy

**Figure 4:**
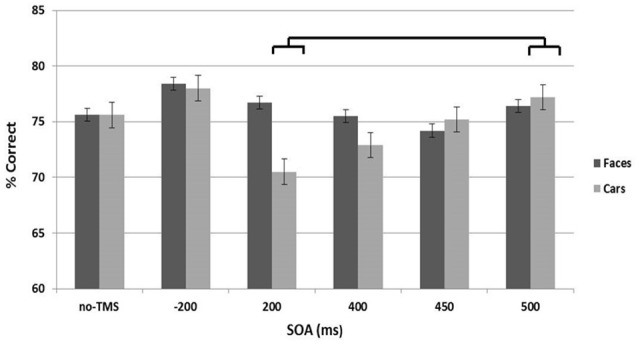
Accuracy across the stimulus onset asynchrony (SOA) conditions collapsed across the left and right stimulation sites are shown separately for the face (dark gray) and car (light gray) stimulus types. Error bars represent standard errors.

The analysis revealed no significant main effects of Site or Stimulus Type (Figure 4). There was, however, a significant main effect of SOA (*F*_(5,8)_ = 3.2, *p* < 0.015) and a significant interaction between Stimulus Type and SOA (*F*_(5,8)_ = 7.4, *p* < 0.01). *Post hoc* *t*-tests indicated a decrease in accuracy at 200 ms for cars, compared to 500 ms SOA (*t*_(12)_ = 2.6, *p* < 0.012; Cohen’s *d* = 0.75).

## Discussion

In this study, we tested the Spatio-temporal model of the neural substrate of visual object decisions identified by Philiastides and Sajda. This model predicted that, at a certain level of perceptual difficulty in discriminating cars from faces (which was controlled on an individual basis), ppTMS to LOC in a predicted time window (i.e., 400–450 ms latency) would interfere with decision-making processes, slowing RT. The results did indeed reveal a significant slowing of RT at the location and latency the Philiastades/Sajda network model expected object discrimination to be occurring, providing support for the model, specifically that LOC plays a role in PDM. Furthermore, we did not find any right or left differences in TMS effects, in line with the relatively equal bilateral LOC activation found by Philiastides and Sajda (2007).

This result is consistent with other studies showing that ppTMS applied to LOC can disrupt visual processing (Ellison and Cowey, 2007; Pitcher et al., 2007, 2008, 2012; Mullin and Steeves, 2011). For example, using an earlier range of SOAs than the current study, Mullin and Steeves (2011) had subjects discriminate natural vs. man-made stimuli, while stimulating LOC with pulse pairs spaced 100 ms apart, and found significant drops in the accuracy (without affecting RT) only at early latencies: 0, 40 and 80 ms with left hemisphere stimulation and 40 ms on the right. Similarly, in a second study, Ellison and Cowey (2007) applied ppTMS spaced 50 ms apart over LOC, at SOAs ranging from 0–350 ms, during a distance discrimination task in which subjects had to choose which of two green squares were closer in proximity to a central green square at fixation. A detrimental effect on performance was also found when TMS was applied at early SOAs of 0, 50, 150 ms with a slowdown of RT compared to sham. This early effect also occurred in a ppTMS study by Pitcher et al. (2008) and dissociated into two separate processes using finer time resolution (paired pulses separated by 10 ms) in Pitcher et al. (2012). Overall, these results suggest that LOC is involved with the early visual processing associated with the the first network in Philiastades/Sajda’s model, although a simpler explanation may be that TMS to LOC at earlier latencies could disrupt processing trans-synaptically *via* feedback connections with regions of the model’s first network active at this time.

By finding effects in a later latency range by using more difficult-to-discriminate object stimuli that required extended PDM processing, the present study not only extends the work done by others who examined the effects of TMS on object processing in LOC, but more importantly, it does so specifically testing a well-developed psychophysiological model of the neural networks involved. The behavioral data from the face/car discrimination task had been modeled by a diffusion drift (or random walk) process (Ratcliff and Rouder, 1998), and fit to psychophysiological data measured at latencies between 300 and 500 ms after the visual stimulus is presented (Philiastides and Sajda, 2006). The Philiastides/Ratcliff/Sajda diffusion model was driven by the accumulation of information, and the cortical interference produced by TMS was expected to disrupt that process during difficult discrimination in a time period beginning at 400 ms, slowing it down by decreasing the drift rate (size of the “steps” taken in the walk). Indeed, the slowest RTs were observed with ppTMS starting at 400 ms SOA, corroborating the hypothesis that the LOC is involved with object decision-making in this time window and providing evidence for the specific model.

There has been a large amount of research on PDM in non-human species primarily involving two, alternative forced-choice (2AFC) tasks in rats and non-human primates in somatosensory, auditory and visual modalities (for reviews, see Gold and Shadlen, 2007; Brody and Hanks, 2016). In general, a number of brain regions have been shown to be involved in processing such tasks. For example, in nonhuman primates, successfully negotiating a tactile frequency-discrimination task has been shown to involve a distributed network including primary and secondary somatosensory cortices, dorsolateral prefrontal cortex, and medial and ventral premotor cortices (Gold and Shadlen, 2007). Visual 2AFC tasks in non-human primates have implicated a number of regions in evidence accumulation and decision making, including prefrontal cortex (Hunt et al., 2012), frontal eye fields (Kim and Shadlen, 1999), posterior parietal cortex (Shadlen and Newsome, 2001), and subcortical areas such as superior colliculus (Horwitz and Newsome, 1999) and basal ganglia (Ding and Gold, 2010), although it is not clear what aspects of decision making each region is responsible for. As Brody and Hanks (2016) point out, despite a great deal of research on the subject, the brain regions underlying the information accumulation process are still unknown. They suggest a research program in which perturbation of a given brain region during task performance may be useful; namely, if a given brain region is involved with decision formation and gradual accumulation of evidence, perturbation should have an effect on task performance, and more specifically perturbation should affect performance in specific time periods (e.g., during the evidence accumulation). Local inactivations using muscimol infusions (Erlich et al., 2015) and optogenetics (Hanks et al., 2015) in rats has provided evidence that the frontal orienting field is involved with premotor output aspects of the decision process, and that posterior parietal cortex may be involved with the accumulation process, although the evidence for that is somewhat equivocal. The present study falls into the Brody and Hanks (2016) framework, with perturbations using TMS to LOC interfering with 2AFC task performance in a specific temporal window. The homolog of human LOC in nonhuman primates is not known, with area V4 or inferotemporal (IT) cortex the leading candidates (Orban et al., 2004). Study of decision processing in nonhuman primates in these areas has not yet been pursued, but the present study in humans suggests V4 or IT may be candidates for the accumulation process in object decision making. Overall, the present study is an example of how temporal- and spatial-specific disturbance of cortical processing using TMS may provide a useful tool to extend animal research in decision processes to humans.

The reduced performance seen at the 200 ms SOA appears to be a qualitatively different TMS effect from the later one (affecting accuracy rather than RT, and being stimulus-specific rather than stimulus-general), suggesting interference of a different kind. The present study was designed to test the third component of the Philiastides et al. (2006) model, and the accuracy effect found at 200 ms was unexpected. In Ellison and Cowey (2007), ppTMS applied to LOC at 200 and 350 ms SOA also diminished performance. The main difference between these two studies and Mullin and Steeves (2011) is the task difficulty. In Mullin and Steeves, the task involved a very easy visual discrimination, as demonstrated by accuracy rates without TMS of 95%, while the discrimination accuracy used in the present study and in Ellison and Cowey (2007) was titrated on an individual basis to be about 75% and 79% correct, respectively. According to Philiastides and Sajda (2006), when discrimination is difficult, a second network exerting top-down influence is activated in the 180–280 ms period, although this the network does not include LOC, at least in terms of fMRI activation. Thus, one possibility is that, with a hard-to-discriminate stimulus, at 200 ms feedforward of information to decision-making areas from LOC indicating no interpretable result activates the network of the second component, and begins the evidence-accumulation process in LOC. The 200 ms TMS may have thus interfered with the influence on the second component and/or the initiation of evidence accumulation in LOC. The fact that only car discrimination accuracy was affected by stimulation at 200 ms might be explained given that the human brain has evolved with dedicated neural architecture for processing face stimuli outside of LOC (e.g., the fusiform face area). These other face-specific areas could help compensate for LOC disruption to maintain accuracy, while car stimuli did not benefit from this dedicated architecture. Thus the disruption at 200 ms is indicative that LOC plays a causal role in earlier sensory processing. On the other hand, the RT slowing with LOC stimulation at 400 ms was not stimulus-specific, suggesting that the additional decision process in the case of difficult object discrimination may require LOC involvement for stimulus categorization in general. Overall, the occurrence of the TMS effect at 200 ms suggests an interaction of the two networks of the second and third components, specifically at LOC, which was not evident in the fMRI but which was brought forth by TMS. This interaction might best be studied using TMS pulses during task performance in the context of concurrent TMS/fMRI in the scanner.

Finally, an unexpected improvement in RT was found when ppTMS was applied at −200 ms SOA, indicating a possible form of perceptual priming. It was anticipated that TMS prior to stimulus delivery would provide a second control time point, since processing of the stimulus would not yet have begun. Despite this expectation, the findings that TMS at −200 ms facilitated performance is consistent with findings of TMS performance facilitation in general (Luber and Lisanby, 2014). For example, one TMS study found that stimulation delivered in the 100 ms before the onset of a target increased its detectability (Grosbras and Paus, 2003). They suggested the TMS potentiated local neural activity for a brief period, noting that in animal studies direct electrical stimulation of neurons in the homologous visual area immediately preceding a target improved performance as well. On the other hand, because TMS also produces superficial effects, a phenomenon known as intersensory facilitation (ISF) may have occurred (Terao and Ugawa, 1997). This facilitation—a speeding of RT—has been shown to occur with TMS pulses applied in the 150 ms period prior to stimulus onset. Thus, further investigation is necessary to determine whether TMS can cause enhancement of visual discrimination processing beyond possible ISF effects, which cannot be distinguished by the design of this study.

While we believe the results of this study demonstrate the usefulness of TMS in validating network models of cortical function, and in particular the Philiastades/Sajda model of visual discrimination, the study did have some limitations that should be mentioned. The sample size was small, leading to a need to replicate the findings in a larger group. TMS coil targeting was done using individual structural MRIs and group-level functional coordinates of task activations, but targeting could be improved by using individual fMRI activations produced by the task. SOAs at which TMS was not expected to affect processing, as well as no-TMS conditions, were used for control comparisons, but the addition of a sham TMS control would have clarified whether the improved performance in the −200 ms condition was due to TMS enhancement or ISF. It should be emphasized that the temporal control used here was not only sufficient to test the visual discrimination aspect of the Philiastades/Sajda model but is an extremely robust form of TMS control to use in this circumstance, where timing within a specific model was tested. Moreover, while the use of sham or active control sites each can be problematic, stimulating the same site at slightly different times works well as a control, as the different conditions stimulate the same nervous tissue and feel the same to the participant, who has little awareness of their difference (with the exception of stimulation occurring immediately before the perceptual stimulus, which can generate ISF). There is a question of whether the addition of a spatial control would have been useful here, in order to distinguish whether the TMS effects observed in the present study were caused by direct cortical stimulation or possibly by the indirect effects of TMS (e.g., auditory and somatic stimulation caused by the TMS coil). As mentioned above, the enhancement effect at −200 ms may have been caused by ISF. There is evidence that indirect effects of TMS can cause performance changes over time beyond pre-stimulus periods: for example, it has been shown that sham TMS can have time-dependent effects (Duecker et al., 2013): in two RT tasks, they found what was most likely the effect of ISF with TMS given prior to visual stimulus onset, but they also found slowing of RT post-stimulus onset which grew with time past stimulus onset. The authors attributed the growing RT to subjects waiting for the TMS pulse before responding to the stimulus. While such an explanation fits the post-stimulus onset data in Duecker et al. (2013), it does not do so with ours. Rather than a smoothly increasing RT across time, we found that TMS did not increase RT with pulses beginning at 200, 450, or 500 ms after visual stimulus onset- they were not significantly different from the no-TMS condition- and instead, TMS in only a single window of time produced slowing. While we acknowledge that TMS can create non-specific effects, to our knowledge there is no report of non-specific effects responsible for such a specific pattern, affecting performance in one time window but not in those immediately around it, in time windows occurring post-stimulus onset. Moreover, we found a second, qualitatively different, effect, this time on performance accuracy at 200 ms, with a significant accuracy decrease, but with no change in RT at that time. As with the case with RT, this was quite time-specific, with accuracy at 400, 450, and 500 ms all matching that in the no-TMS condition, and to our knowledge there are no reports of accuracy changes attributable to non-specific effects post- stimulus onset, especially in a single-window between other unaffected windows, nor do we believe there are reports of two qualitatively different TMS effects occurring in single separate windows of time within the same blocks of task trials that were attributable to non-specific effects. This was a first attempt to test the model of Philiastides et al. (2006) and certainly more work, with more controls and tests, needs to be done. It is possible that the combination of TMS effects we observed could somehow be due to non-specific effects, but given the specific pattern observed, it seems to us to be unlikely.

Future studies examining the Philiastades/Sajda model should also take account of recent findings regarding regions in LOC specialized for particular types of stimuli such as faces in choosing task stimuli and specific cortical targets. Philiastides and Sajda (2007) found that LOC activation for cars was located slightly dorsally to the activation for faces. More recent work using imaging and TMS supports the idea that LOC may be subdivided into a number of regions that specialize in different sorts of visual stimulus categories (Pitcher et al., 2012). In interpreting the present results, an additional area (or areas) devoted to face categorization and identification beyond LOC might explain why only car discrimination accuracy was affected by stimulation at 200 ms. Under this logic, other face-specific areas could help compensate for LOC disruption to maintain accuracy while identifying face stimuli. On the other hand, the RT slowing with LOC stimulation at 400 ms was not stimulus-specific, suggesting that the additional decision process in the case of difficult object discrimination may require LOC involvement for stimulus categorization in general.

## Conclusion

This study represents a first step in using TMS to verify an established multiple-network model of visual object discrimination, which was based on psychophysical, EEG and fMRI measurements taken during a challenging discrimination task. We were able to provide causal evidence for a prediction of the model that TMS applied to LOC at 400 ms would slow RT. In addition, we were able to observe other effects caused by TMS, notably a potential performance enhancement, that could be interpreted by the model and which lead to future experiments using TMS to engage the three networks posited by the model and explore their interactions. Further studies, especially using simultaneous TMS/fMRI to observe the immediate and long-range effects of TMS within the posited networks, provide exciting possibilities to extend this research.

## Data Availability Statement

The datasets generated for this study are available on request to the corresponding author.

## Ethics Statement

This study was carried out in accordance with the recommendations of Institutional Review Board of the Duke University Medical Center with written informed consent from all subjects. All subjects gave written informed consent in accordance with the Declaration of Helsinki. The protocol was approved by the Institutional Review Board of the Duke University Medical Center.

## Author Contributions

BL contributed to experimental design, supervised MRI and TMS testing and data analysis, and prepared the manuscript. DJ contributed to experimental design, developed the cognitive testing, carried out MRI and TMS testing and data analysis, and revised the manuscript. GA contributed to data analysis and revised the manuscript. AH contributed to experimental design, carried out MRI and TMS testing, and revised the manuscript. SH and LB contributed to data analysis and revised the manuscript. TJ contributed to experimental design, carried out MRI and TMS testing, and revised the manuscript. PS initiated and contributed to the experimental design, helped create the cognitive tasks and testing, and revised the manuscript. SL contributed to experimental design, provided access to TMS equipment and facilities, and revised the manuscript.

## Conflict of Interest

The authors declare that the research was conducted in the absence of any commercial or financial relationships that could be construed as a potential conflict of interest.

## Footnotes

^1^http://faces.kyb.tuebingen.mpg.de
